# Challenges in Exosome Isolation and Analysis in Health and Disease

**DOI:** 10.3390/ijms20194684

**Published:** 2019-09-21

**Authors:** Nils Ludwig, Theresa L. Whiteside, Torsten E. Reichert

**Affiliations:** 1 Department of Pathology, University of Pittsburgh School of Medicine, Pittsburgh, PA 15213, USA; ludwign@upmc.edu (N.L.); whitesidetl@upmc.edu (T.L.W.); 2 UPMC Hillman Cancer Center, Pittsburgh, PA 15213, USA; 3 Departments of Immunology and Otolaryngology, University of Pittsburgh School of Medicine, Pittsburgh, PA 15213, USA; 4 Department of Oral and Maxillofacial Surgery, University Hospital Regensburg, 93053 Regensburg, Germany

**Keywords:** exosomes, extracellular vesicles (EVs), exosome isolation, biomarkers, drug delivery, tumor-derived exosomes (TEX)

## Abstract

A growing body of evidence emphasizes the important role exosomes in different physiological and pathological conditions. Exosomes, virus-size extracellular vesicles (EVs), carry a complex molecular cargo, which is actively processed in the endocytic compartment of parental cells. Exosomes carry and deliver this cargo to recipient cells, serving as an intercellular communication system. The methods for recovery of exosomes from supernatants of cell lines or body fluids are not uniformly established. Yet, studies of the quality and quantity of exosome cargos underlie the concept of “liquid biopsy.” Exosomes are emerging as a potentially useful diagnostic tool and a predictor of disease progression, response to therapy and overall survival. Although many novel approaches to exosome isolation and analysis of their cargos have been introduced, the role of exosomes as diagnostic or prognostic biomarkers of disease remains unconfirmed. This review considers existing challenges to exosome validation as disease biomarkers. Focusing on advantages and limitations of methods for exosome isolation and characterization, approaches are proposed to facilitate further progress in the development of exosomes as biomarkers in human disease.

## 1. Introduction

Exosomes are virus-size membranous vesicles ranging in the diameter from 30–150 nm. They originate from the endocytic compartment of the producer cell. A growing body of evidence indicates that exosomes play a major role in intercellular communication in physiological as well as pathological conditions [1]. The rapidly increasing number of publications emphasizes the growing interest in explorations of biologic functions of exosomes. Compared to microvesicles (MVs), which are secreted by “budding” or “pinching off” from the cell membrane surface, the biogenesis of exosomes is more complex. It involves reverse membrane invagination and processing in multivesicular bodies (MVBs), followed by exosome release into intercellular fluid when MVBs fuse with the cell membrane [2]. Due to the endosomal origin, exosomes are enriched in late endosome components such as CD63, CD9 and CD81 [3]. The complex biogenesis suggests that exosomes, whose cargo reflects the molecular processing taking place inside the parent cell, are best suited to serve as potential surrogates of this parent cell in body fluids. The fidelity of exosome cargo with that of the parent cell has been confirmed in several studies [4]. Analysis of exosome levels in body fluids of patients and of exosome cargos emerges as a potential diagnostic tool of disease or predictor of disease progression, response to therapy and overall survival [5,6,7]. Since exosomes can be isolated from all body fluids, they are optimal candidates for analysis as part of a non-invasive liquid biopsy [8]. A therapeutic application of exosomes has also been suggested for different diseases, utilizing their biological activity and the rich cargo they carry to serve as potential cell-free therapy [9]. Exosomes have been implicated in drug resistance and in reprogramming of tissue environments in various diseases [10,11,12]. Universal exosome involvement in cellular interactions creates an urgent need for a better understanding of their characteristics and functions. Hence, the isolation and molecular/genetic analyses of exosomes are currently a priority.

Studies of body fluid-derived exosomes are a challenge, since the isolated exosomes are derived from many different cell types and contain components of body fluids (“contaminants’), which have to be removed during exosome isolation. Most of what is currently known about exosomes comes from studies of cell lines propagated in long-term cultures. This offers an opportunity of examining exosomes derived from a single, well-defined cell type [13]. However, recent technological progress in exosome isolation and their analysis has not led to a universally accepted recommendation for exosome isolation. The field is facing problems in selecting and reproducing various isolation methodologies. Additionally, approaches to data acquisition/analysis vary widely. The recommendations formulated in the guidelines of the International Society for Extracellular Vesicles (ISEV) are vague and leave extracellular vesicles (EV) isolation to investigators’ discretion [14]. This situation reflects the young age of the fast-developing EV field and the recommendations by ISEV are produced as a community effort which involves the leading EV researchers. The guidelines go on to note that some major conclusions in published literature are often insufficiently supported by the reported experimental evidence. For these reasons, the guidelines charge the investigators to closely specify the utilized methodologies presumably to accumulate the described data for formulating future recommendations [14].

Not only exosome isolation methods, but also the nomenclature of EVs remains unclear. The recent introduction of exomeres (<30 nm in diameter), in addition to small and large exosomes, all with potentially similar biogenesis, and larger EVs (>200 nm), encompassing microvesicles, oncosomes and apoptotic bodies, emphasizes the heterogeneity of EVs [15]. Recognizing the existing uncertainty and confusion in the EV field, we highlight and summarize the challenges facing exosome research today. The focus is on the methodologies for exosome isolation, the influence of cell culture parameters on the quantity and quality of isolated exosomes and the consequences of different treatments of the parental cells on exosome production. The current technologies for the analysis of the exosome cargo and their limitations will be discussed and solutions for improvements will be considered.

## 2. Influence of Exosome Isolation/Purification Methodologies on the Composition of Exosome Cargos

In the field of EV research, no gold standard for exosome isolation exists, and new methodologies seem to appear almost daily. Ultracentrifugation (including differential centrifugation) remains by far the most widely used primary isolation method for EVs, being used by more than 80% of all EV researchers worldwide [16]. Although ultracentrifugation is widely used in the field, the technique has several drawbacks, including the co-isolation of non-exosomal impurities, low reproducibility, low RNA yield, potential damage of exosomes and low-throughput of samples, which is not compatible with clinical utilization [17]. Investigators who exclusively use conditioned culture media for exosome isolation mostly use ultracentrifugation, whereas those studying complex biological fluids, such as plasma, tended to use a combination of different methods for isolation and purification of EVs. Among these methods are density gradient centrifugation at lower speeds to remove cell fragments and large platelet-derived vesicles, followed by ultrafiltration and size-exclusion chromatography (SEC) [16]. Each of the multiple isolation methods available today has advantages and disadvantages and absolute exosome purity remains an unrealistic goal that is only partially met by methods such as SEC, which removes considerable (but not all) quantity of “contaminating’ plasma or medium components [13]. Recently, Lyden’s group introduced a new method for EV isolation based on asymmetric-flow field-flow fractionation (AF4). Using AF4, nanoparticles are separated based on their density and hydrodynamic properties by two perpendicular flows, i.e., the forward laminar channel flow and the variable crossflow. This method allowed for the identification of exomeres as well as two exosome subpopulations that demonstrate distinct biophysical and molecular properties [15]. This and similar discoveries will accelerate the successful performance of translational studies mediated by EV subsets in diagnostic, prognostic and therapeutic applications.

The method used for EVs isolation from body fluids is critical for the characterization of their cargo. Reports in the literature suggest that different isolation methods introduce variations in the concentration, purity and size of exosomes [18,19,20]. Exosomes isolated by ultracentrifugation, ExoQuick or Total Exosome Isolation Reagent yielded mRNA that showed differences upon subsequent RNA sequencing analysis. Although 588 common miRNAs were found in all three exosome samples, each sample isolated by the different method contained approximately 200 miRNAs, which were unique for each exosome preparation [18]. Ding et al. reported similar results showing that the exosomal miRNA profiles are distinct, depending on the isolation methods used [21]. Additional evidence was provided by Rekker et al., who also concluded that the miRNA profile in exosomes may be altered by the isolation method [22]. Proteomic or flow cytometry-based analyses of exosomes yielded similar results, suggesting that isolated exosome fractions are often “contaminated” by co-isolated plasma proteins and that the extent of contamination depends on the isolation method used [16]. Common co-isolated soluble proteins are albumin, immunoglobulins and matrix metalloproteases (MMPs), all of which are abundantly present in body fluids [23,24]. The available data lead to the conclusion that approaches to exosome isolation from plasma or body fluids remain the most critical aspect for the characterization of exosome molecular or genetic cargos. Thus, it is necessary to proceed with caution in selecting the appropriate purification method for EVs, and to carefully discriminate between the true exosome content versus “contaminating” plasma proteins or nucleic acids coating the exosome surface.

Isolation of exosomes by direct immunocapture from cell culture supernatants or body fluids might overcome the ”contamination” problem; however, immune capture is strictly dependent on specific binding of the detection antibody to an antigen on the exosome surface. Therefore, the presence of “contaminants” is likely to interfere with immune capture. We have reported that immunocapture of exosomes after their isolation from plasma by, e.g., size exclusion chromatography (SEC), which eliminates most, but not all, of contaminants, might reduce the interference by non-EV-associated proteins [25]. The use of mass spectrometry (LC-MS/MS) for detection of exosome-associated proteins is widely used. However, it is important to acknowledge that mass spectrometry might provide artefactual results due to contaminations of exosomes with high-abundance plasma proteins that can mask low-abundance exosome-specific components [26]. Various functional studies performed with plasma-derived exosomes also showed that functions of the isolated exosomes may be negatively influenced by “contaminating”, non-vesicular materials [23].

Furthermore, comparisons of different isolation techniques indicated that altered levels of several proteins widely used as “markers” of exosomes, such as tetraspanins, varied among different isolation techniques [27] and between exosomes derived from different tumor cells [13]. Exosome markers may be differential due to different EV isolation methods producing different purity, but also because they may isolate different subpopulations of EVs. Similar results were also observed for angiogenic factors, which were present at significantly different levels in a study comparing ultracentrifugation and SEC [23]. The reason for these altered levels of protein “markers” may again be due to the ratios of EV/non-EV components in the cargo of the isolated exosomes.

In aggregate, the “purity” of exosome samples is highly dependent on the vesicle isolation method, and it is likely to affect results interpretation. However, highest purity usually comes in expense of low yield and for some biomarker studies, the purity, i.e., where the biomarker came from (EVs/co-isolating matrix), may not matter if the biomarker is reproducibly detected in the EV preparations. For some applications, such as proteomic analyses, it is possible that the analyses of exosome cargo might not be able to identify proteins present in femtomolar quantities in exosomes, especially in the presence of “contaminating” proteins. The solution may be amplification of these proteins using monoclonal antibodies, which will detect the ‘rare’ exosome components with exquisite specificity, as we and others have demonstrated [28].

## 3. Culture Conditions Influence Exosome Levels and Cargo

The cell culture conditions used for exosome production vary greatly among laboratories, even when well-established cell lines are used. The yield and cargo of exosomes can be drastically altered by culture conditions used for producer cell lines. Finding the optimal conditions for exosome production by a specific cell type remains a challenge, since it is always a compromise between optimal conditions for growth and those for exosome production and isolation. Different parameters of cell culture can influence the cargo composition of exosomes as illustrated below.

### 3.1. Culture Containers

Comparisons of exosomes derived from cells cultured in conventional cell culture dishes versus two-chamber bioreactors indicated that the morphology, size distribution and surface markers of exosomes were similar. However, the yield of exosomes was more than 100 times higher in bioreactors than in dishes, and the metabolomic analysis revealed statistically significant differences [29].

### 3.2. Cells

Each cell type requires unique medium composition for optimal growth and unique seeding density, passage frequency and medium replacement. As alluded to already, the requirements for cell growth have to be harmonized with the requirements for the exosome isolation and optimized for the intended downstream applications [26]. A prerequisite for any EV-related study is the use of contamination-free cells. Mycoplasma or other microbes can also release EVs interfering with the results of exosome characterization, cargo analysis or functional studies [30,31,32].

The passage number can impact the cargo composition and functions of exosomes, as shown for mesenchymal stromal cells (MSCs). Within five serial passages, the level of the exosome “marker” CD63 was reduced, and the recovered exosomes were less potent in stimulating migration of endothelial cells [33]. Umezu et al. compared exosomes derived from bone marrow stromal cells of normal human donors and reported that exosomes derived from older donors showed significantly lower functions than those derived from younger donors [34]. Cells maintained in long-term cultures and undergoing numerous passages are likely to differ in exosome production.

Numbers of cells seeded to initiate cultures are very important for exosome production and their “purity”. It has been reported for several different cell lines that 36h of subconfluency and 36h of confluency during a 72h incubation period resulted in sufficient exosome yields and reduced “contaminations.” Seeding higher cell numbers decreased the levels of exosomes per cell and increased contamination with medium-derived proteins [13]. Besides regulating the sample purity, the use of different cell numbers can have a direct impact on the cargo and functions of exosomes, as shown by Patel et al. [33]. Therefore, to guarantee the reproducibility and constancy of levels and cargo of the isolated exosomes, it is necessary to establish and utilize the same cell seeding protocol. Using a similar passage of producer cells further ensures the constancy of exosome quality [13].

### 3.3. Medium Composition

The medium used for cell culture and exosome recovery from supernatants emerges as a crucial factor in exosome production. All medium components influence the production and/or composition of exosomes; this emphasizes the importance of choosing the right components and reporting them in the publications. The medium composition has to be harmonized with the intended vesicle use and desired exosome “purity”. The medium that best supports growth of producer cells and yields exosomes with a reproducibly stable cargo should be selected and consistently used for culture of exosome producer cell lines.

The levels of glucose used in culture media play an especially important role in exosome production. Burger et al. demonstrated that high glucose levels enhanced EV production and promoted secretion of bigger vesicles sized at 250 nm. A proteomic exosome analysis indicated that the high glucose content significantly altered the molecular composition of EVs, resulting in the presence of exosome proteins, which induced activation of molecular pathways in recipient cells that differed from those induced by EVs derived from cells cultured with lower glucose levels [35]. Similar results were reported by Rice et al., who also measured increased exosome production under high glucose and described an altered bioactivity of exosomes derived from high versus low glucose conditions [36]. On the other hand, glucose starvation also increased exosome secretion and changed the protein content. Gene Ontology (GO) analysis demonstrated different biological processes associated with the protein pattern found in exosomes from conditioned medium with or without glucose starvation [37].

Antibiotics are another widely used component of culture media. It was shown that ciprofloxacin, which is broadly used in cell cultures to control mycoplasma contamination, increases the level of DNA, which is associated with exosomes and is carried on the surface of the vesicles. Exosomes derived from ciprofloxacin-treated cells showed a DNA-dependent binding to fibronectin [38].

Of major importance are medium components, which are highly enriched in proteins and contain EVs, such as fetal bovine serum (FBS). Exosomes present in FBS were reported to be biologically-active, and their co-isolation might lead to interference in subsequent in vitro or in vivo studies [39]. Thus far, no standardized protocol for the exosome-depletion of FBS exists, and different research groups use various approaches to depletion of bovine exosomes from sera. One approach is to culture cells in in serum-free medium during the exosomes-release period; however, the missing FBS can lead to extensive starvation of the cells, can change the cellular behavior and the composition of secreted exosomes [26,40]. Although cells cultured in serum-free media did not result in EVs with significantly different biophysical or size differences as compared with cells cultured in serum-containing media, the quantity of isolated EVs was greatly altered. Moreover, the levels of certain vesicular proteins (e.g., small GTPases, G-protein complexes, mRNA processing proteins and splicing factors) were found to be different in EVs produced under different culture conditions [41]. The use of commercial exosome-depleted FBS is another alternative. However, the manufacturer usually does not describe the method used for exosome depletion. Therefore, the ISEV guidelines recommend the utilization of home-generated depletion protocols instead, provided the protocol used is described in detail [14]. A variety of protocols are available for exosome depletion of FBS; most involve extensive ultracentrifugation or filtration [42,43]. These protocols may result in a loss of growth factors present in FBS, decreasing its growth-promoting effects. Since a full depletion of EVs may not be possible, the ISEV guidelines recommend the use of fresh media not cultured with cells as controls in downstream exosome assays [14]. Specific miRNAs abundant in FBS, such as miR-122, miR-451a and miR-1246, have been previously reported to be enriched in cell culture-derived EVs, emphasizing the potentially confounding effect of the FBS on results of highly sensitive gene expression profiling technologies [44]. In addition, not only FBS in culture supernatants but also proteins present in serum or plasma or any body fluid used as a source of EVs can “contaminate” exosomes leading to artefactual data in downstream applications, especially in mass spectrometry [26].

Since the exosome isolation from conditioned media containing FBS can lead to the co-isolation of exogenous EVs, it was suggested that cells can be cultured without serum using platelet lysates, pituitary extracts, bile salts or synthetic factors instead [14]. However, growth factors might exert regulatory effects on the exosome release. For example, Zhou et al. investigated the role of EGF on exosome production [45]. They reported that the production was significantly decreased in cells treated with EGF, but it increased after treatment of the cells with gefitinib (EGFR inhibitor).

### 3.4. Hypoxia

Hypoxia is an important environmental factor that affects exosome production. It was shown, that the exosome secretion is elevated under hypoxic conditions [13,46]. Moreover, hypoxia altered the exosome cargo largely by changing the oxygenation status of the producing cells [4]. The levels of exosomal markers such as tetraspanins can be altered under hypoxic conditions as recently described [13]. Additionally, several exosomal miRNAs were found to be up-regulated. For example, exosomes secreted by cardiosphere-derived cells under hypoxia had increased levels of pro-angiogenic miRNAs such as miR-126, miR-130a and miR-210 [47]. Kucharzewska et al. described several pro-angiogenic proteins, which were carried at higher levels by exosomes derived from hypoxic cells. The hypoxia-derived exosomes also had higher activity in the in vitro and in vivo angiogenesis assays [4].

### 3.5. Treatments/Perturbations of Cells

It has been reported that the formation of MVBs and the release of exosomes by producer cells is dependent on the intracellular calcium concentration. Monensin, which is a Na^+^/H^+^ exchanger that induces changes in intracellular calcium, was shown to stimulate exosomes release in a concentration-dependent manner [48]. In contrast, GW4869, a neutral sphingomyelinase 2 inhibitor, was shown to block exosome release in vitro and in vivo [49,50]. Another factor, which modulates exosome release, is heparanase. The expression of this enzyme is upregulated during tumor progression and enhanced heparanase expression or exposure of cells to exogenous heparanase stimulates exosome secretion. Interestingly, heparanase also modulates the exosome cargo composition, as seen by an enrichment of syndecan-1, VEGF and hepatocyte growth factor in exosomes and an increased biological activity in functional studies [51]. Datta et al. used a high-throughput screen to identify compounds which directly modulate exosome biogenesis in vitro. Testing a total of 4580 pharmacologically active compounds, they identified 22 compounds that either stimulated or reduced exosome production. The authors validated the lead compounds tipifarnib, neticonazole, climbazole, ketoconazole and triademenol as potent inhibitors and sitafloxacin, forskolin, SB218795, fenoterol, nitrefazole and pentetrazol as activators of exosome production [52]. These findings indicate that some of the commonly used drugs could be re-utilized for targeting the exosome biogenesis and may also have the potential to modulate the cargo composition of exosomes.

Oxidative stress has also been described as a stimulator of exosome production. Atienzar-Aroca et al. applied oxidative stress to cells by exposing them to increasing concentrations of ethanol. The cellular response was a two-fold increase of exosome production, and the exosomes carried higher levels of VEGFR in the membrane as well as an extra cargo of VEGFR mRNA in the lumen [53].

Hyperthermia represents another factor, which alters exosome composition and levels. Exosomes derived from heat-stressed tumor cells were enriched in chemokines, such as CCL2, CCL3, CCL4, CCL5 and CCL20 and attracted immune cells in vitro and in vivo [54].

In summary, exosome production by cells and exosome molecular content appear to be regulated by a variety of factors and conditions. Cultured cell lines represent an important resource for studies of exosomes. However, EVs, including exosomes, show a high degree of plasticity in their cargo and the molecular and genetic analyses of EV-associated components remain challenging.

As in vitro production of exosomes by cell lines is strictly dependent on the producer cell growth and metabolism, the utilization of pre-tested, standardized cell culture conditions is a necessary requirement for obtaining sufficient and reproducible exosome preparations.

## 4. Disease-Associated Alterations in Exosome Production/Cargo

The levels and cargos of exosomes produced by cultured cells can be readily modulated by environmental factors or pharmacological treatments. It is thus reasonable to expect that levels and molecular cargos of exosomes isolated from blood or other body fluids of patients with various diseases might be altered having been produced by diseased cells.

Indeed, numerous studies have shown that patients with different types of cancer have elevated levels of circulating exosomes in their blood [46]. The presence of tumor-specific proteins in the cargo of circulating exosomes confirms that these exosomes are derived from tumor cells [55]. The protein profile of tumor-derived exosomes (TEX) shows a high degree of plasticity and is likely modulated by cancer therapeutics and/or by cargo alterations occurring during disease progression [6]. Similar observations have been made for other diseases such as HIV. Levels of plasma exosomes were increased in HIV-positive patients compared to healthy donors, and plasma-derived exosomes of HIV patients carried proteins related to immune activation and oxidative stress [56]. Interestingly, the treatment with antiretroviral drugs increased exosomes production and altered their content [57]. It has also been established that the onset of critical illnesses, such as acute lung injury, acute kidney injury, acute myocardial injury or sepsis, is associated with increased levels and altered molecular profiles of circulating exosomes. In nearly all of these diseases, specific exosome-associated proteins or miRs have been identified as potential disease biomarkers [58]. An example is provided by endothelial cell-derived exosomes, which carry high levels of atherosclerosis-promoting proteins and thus have been considered as diagnostic biomarkers for patients with cerebrovascular disease [59].

### Effects of Cancer Therapeutics on EV Production, Content and Function

A growing body of evidence suggests that novel as well as conventional cancer therapies exert profound impact on exosome secretion and their cargo composition [6,60]. These studies indicate that exosome levels as well as their cargos might be utilized to predict responses to therapy and even outcome. Thus, plasma exosomes have a potential to serve as predictive biomarkers in cancer. Recently, Keklikoglou et al. demonstrated that cytotoxic chemotherapy in breast cancer enhances the production of exosomes, upregulates the levels of annexin A6 in exosomes and promotes a pre-metastatic niche formation caused by exosomes [60]. Similar findings were reported by Bandari et al. who demonstrated that chemotherapy-induced exosomes have a proteome profile that is distinct from that in cells not exposed to the drug. The authors showed that exosomes secreted by cells undergoing chemotherapy have dramatically altered functions and promote chemoresistance, tumor survival and progression [61]. Monitoring exosomes derived from the plasma of patients with acute myeloid leukemia (AML) indicated that the patients have high levels of TGF-β1 on exosomes at diagnosis, that these levels decrease after induction chemotherapy and increase during consolidation chemotherapy. The exosome levels remained elevated in AML patients whose leukemia recurred, and they normalized in patients in long-term remission [6].

Other environmental stress conditions, including ionizing radiation, also influence exosome secretion. Exosomes derived from irradiated cells can transfer radiation-mediated signals to other cells, transferring radiation-related effects to the recipient cells, which have not been exposed to radiation [62]. Exosomes derived from irradiated cells also show an altered behavior in functional studies: they promote survival of irradiated cells [63]. Mechanistically, it was shown that irradiation induces pathways of exosome secretion regulated by TSAP6, which is transcriptionally regulated by p53. Thus, the rate of exosome secretion and exosome composition are influenced by DNA damage caused by irradiation [62]. Abramowicz et al. reported that the cargo of exosomes produced by head and neck cancer cells was altered by ionizing radiation. The exosomes produced by the treated cells contained 472 proteins, whose abundance was significantly affected by radiation delivered at different doses. Most of these proteins were upregulated and only 47 proteins were downregulated [64]. Low-level laser irradiation (LLLI) was described to influence angiogenesis in vitro and in vivo. Bagheri et al. showed that LLLI at high power density directly affected endothelial cells by promoting exosome release [65]. This finding indicates that the ratio of tumor cell-derived to endothelial cell-derived exosomes in the TME and/or in the blood might be modulated by LLLI.

## 5. Conclusions

The process of exosome packaging and production in the endocytic compartment of the producer cell is characterized by substantial plasticity, which is mainly modulated by stress-related environmental factors. The analysis of the exosome cargo emerges as a promising diagnostic/prognostic tool and is of great current interest, because exosomes mimic the content of parental cells in their environment. However, no “gold standard” for exosome isolation exists, and the exosome nomenclature remains undefined. Validation of promising findings reported for exosomes by numerous investigators is not possible until a uniformly defined methodology for their isolation and characterization is in place. This is a major challenge facing the field, and while it is being addressed by introduction and evaluation of novel technologies, the results of studies with EVs, including exosomes, remain open to criticism. It is to be expected that the existing limitations in studies of exosomes will soon be resolved and will confirm the roles exosomes play as messengers of intercellular signaling and as biomarkers of impending disease states.
